# Gene expression profiling of the green seed problem in Soybean

**DOI:** 10.1186/s12870-016-0729-0

**Published:** 2016-02-01

**Authors:** Renake N. Teixeira, Wilco Ligterink, José de B. França-Neto, Henk W.M. Hilhorst, Edvaldo A. A. da Silva

**Affiliations:** Wageningen Seed Lab, Laboratory of Plant Physiology, Wageningen University, Droevendaalsesteeg 1, Wageningen, 6708 PB The Netherlands; Departamento de Produção e Melhoramento Vegetal, Faculdade de Ciências Agronômicas-UNESP, Universidade Estadual Paulista, Botucatu, SP 18.610-307 Brazil; Empresa Brasileira de Pesquisa Agropecuária, Centro Nacional de Pesquisa de Soja, EMBRAPA Soja, Caixa-postal 231, Londrina, PR 86001970 Brazil

**Keywords:** Chlorophyll retention, Differential expression, Drought stress, Green seeds, Heat stress, Seed quality

## Abstract

**Background:**

Due to the climate change of the past few decades, some agricultural areas in the world are now experiencing new climatic extremes. For soybean, high temperatures and drought stress can potentially lead to the “green seed problem”, which is characterized by chlorophyll retention in mature seeds and is associated with lower oil and seed quality, thus negatively impacting the production of soybean seeds.

**Results:**

Here we show that heat and drought stress result in a “mild” stay-green phenotype and impaired expression of the *STAY-GREEN 1* and *STAY-GREEN 2* (*D1*, *D2*), *PHEOPHORBIDASE 2* (*PPH2*) and *NON-YELLOW COLORING 1* (*NYC1_1*) genes in soybean seeds of a susceptible soybean cultivar. We suggest that the higher expression of these genes in fully mature seeds of a tolerant cultivar allows these seeds to cope with stressful conditions and complete chlorophyll degradation.

**Conclusions:**

The gene expression results obtained in this study represent a significant advance in understanding chlorophyll retention in mature soybean seeds produced under stressful conditions. This will open new research possibilities towards finding molecular markers for breeding programs to produce cultivars which are less susceptible to chlorophyll retention under the hot and dry climate conditions which are increasingly common in the largest soybean production areas of the world.

**Electronic supplementary material:**

The online version of this article (doi:10.1186/s12870-016-0729-0) contains supplementary material, which is available to authorized users.

## Background

Chlorophyll (Chl) retention in mature soybean seeds is a major problem for Brazilian soybean growers. If more than 9 % [1] of their harvest shows this phenotype, marketability will be compromised, resulting in serious financial losses. The “green seed problem” has been reported in oilseeds since the early 1990s, i.e. as detrimental to soybean seed and oil quality [1–3] and to canola oil quality [4–7].

Retention of Chl in soybean seeds is mainly associated with low rainfall and high temperatures during the maturation phase. These are common environmental conditions in tropical areas such as the Brazilian cerrado biome where approximately 45 % of the Brazilian soybean seed production is concentrated (14.5 million hectares) [8]. Besides the climate, other factors or practices during pre- and post-harvesting, such as application of desiccants and premature harvesting followed by drying at high temperatures are also reported to result in retention of chlorophyll in soybean seeds [2]. In addition to these environmental factors there is also variation in the susceptibility of soybean cultivars to green seed production under stress. The cultivar BRS 133 has been previously described as tolerant, producing less green seeds under stressful conditions whereas MG/BR 46 was found to be more susceptible [9].

During leaf senescence, the first observable change is yellowing of green tissues. Yellowing is also observed during normal seed maturation which is expected to be a degreening process resulting from the conversion of Chl into colourless products in a multi-step catabolic pathway similar to that described for leaf senescence [10–12]. Leaf senescence has been extensively studied and mutant lines exhibiting altered leaf senescence have been described for several different species e.g. Arabidopsis, rice, Medicago and soybean. These mutant lines are commonly known as *stay green* (SGR) mutants [13–25]. However, in contrast to leaf senescence, very little is known about Chl degradation in developing and maturing seeds. Degreening in maturing seeds cannot be considered senescence but both leaf senescence and seed maturation require Chl degradation for their completion.

In leaves Chl degradation occurs in the chloroplast and requires six known Chl-catabolic enzymes (CCEs) and a metal-chelating substance (MCS) that remains to be identified [26]. The breakdown starts with the two-step reduction of Chl b to Chl a, catalysed by Chl b reductase and 7-hydroxymethyl Chl *a* reductase (HCAR) [27]. In the next step the central Mg^2+^ ion of Chl is removed by an unknown MCS to yield a Mg-free Chl intermediate known as pheophytin *a* (Phein *a*). Subsequently, pheophytinase (PPH), catalyses the hydrolysis of Phein *a* to produce pheophorbide a (Pheide *a*) [22, 28]. The chlorin macrocycle of Pheide *a* is oxygenolytically opened by Pheide *a* oxygenase (PAO) [14], and the product of this reaction, red Chl catabolite (RCC), is reduced to a non-phototoxic primary fluorescent Chl catabolite (pFCC) by RCC reductase (RCCR) [29]. Thereafter, pFCC is transferred to the cytosol and stored in vacuoles as non-fluorescent Chl-catabolite [10, 11, 20, 26]

In addition to CCEs and MCS, the SGR protein acts as a key regulator of Chl degradation. *SGR* is a nuclear gene encoding a chloroplast targeted protein, and its homologs exist as either single or duplicated genes in higher plants [16]. Most higher plant species have two or more SGR homologs (STAY-GREEN – SGR and STAY-GREEN LIKE – SGRL), all of which are predicted to localize to the chloroplast [16,30]. In a recent study, phylogenetic analysis suggested that the soybean genes, Glyma11g02980 and Glyma01g42390, are homologs of *PsSGR*, *AtNYE1*, *AtNYE2*, and *OsSGR* [17]. These genes were named *D1* (Glyma01g42390) and *D2* (Glyma11g02980) and described in soybean as two unlinked, paralogous nuclear genes, whose double-recessive mutant (*d1d1d2d2*) results in Chl retention in soybean leaves and seeds [17].

The increase of environmental stress over the past decades is a growing concern worldwide especially for agricultural productivity. Efforts have already been made to understand the influence of growing conditions and drying processes on the retention of green pigments in soybean seeds and how it affects the physiological and biochemical characteristics of these seeds [2, 3, 9, 31, 32]. So far, the molecular mechanisms of Chl degradation in seeds and consequently of Chl retention remain unsolved. In this study we have used maturing seeds of two soybean cultivars differing in susceptibility to stress-induced Chl retention to investigate the molecular basis of chlorophyll retention in seeds. A global gene expression approach was performed in association with targeted gene expression analysis (RT-qPCR) to demonstrate that the green phenotype in stressed soybean seeds is related to impaired expression of chlorophyll catabolic genes (CCGs), stay-green genes and also of some photosynthetic proteins. Our results contribute both to the understanding of the physiological underpinning of Chl degradation in seeds and to the identification of possible breeding targets for future soybean improvement.

## Results

### Chlorophyll content in maturing soybean seeds

**Fig. 1:**
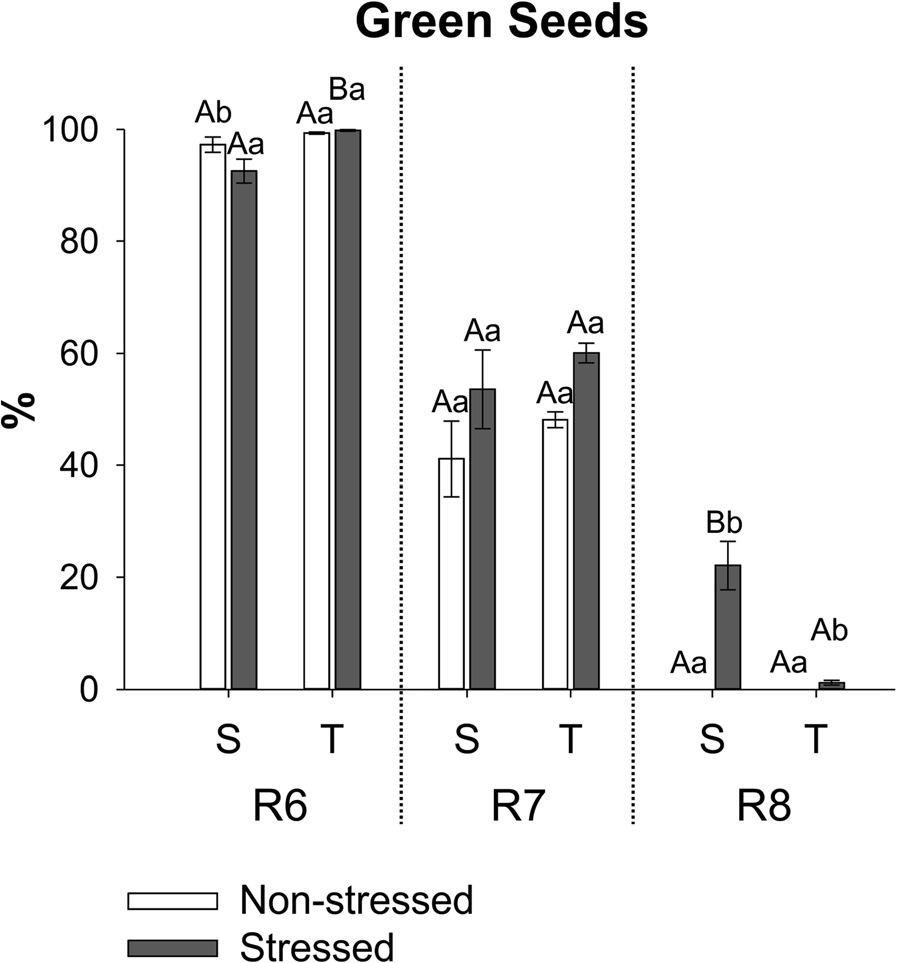
Percentage of green seeds produced under non-stressed (open bars) and stressed (closed bars) environmental conditions by the susceptible (S) and tolerant (T) cultivars in three stages of maturation (R6-R7-R8). Bars represent the mean values ± SE of six independent biological replicates. Lowercase letters represent statistically significant differences (P ≤ 0.05) between different environmental conditions within the same cultivar and developmental stage and uppercase letters between different cultivars within the same environmental condition and developmental stage

**Fig. 2:**
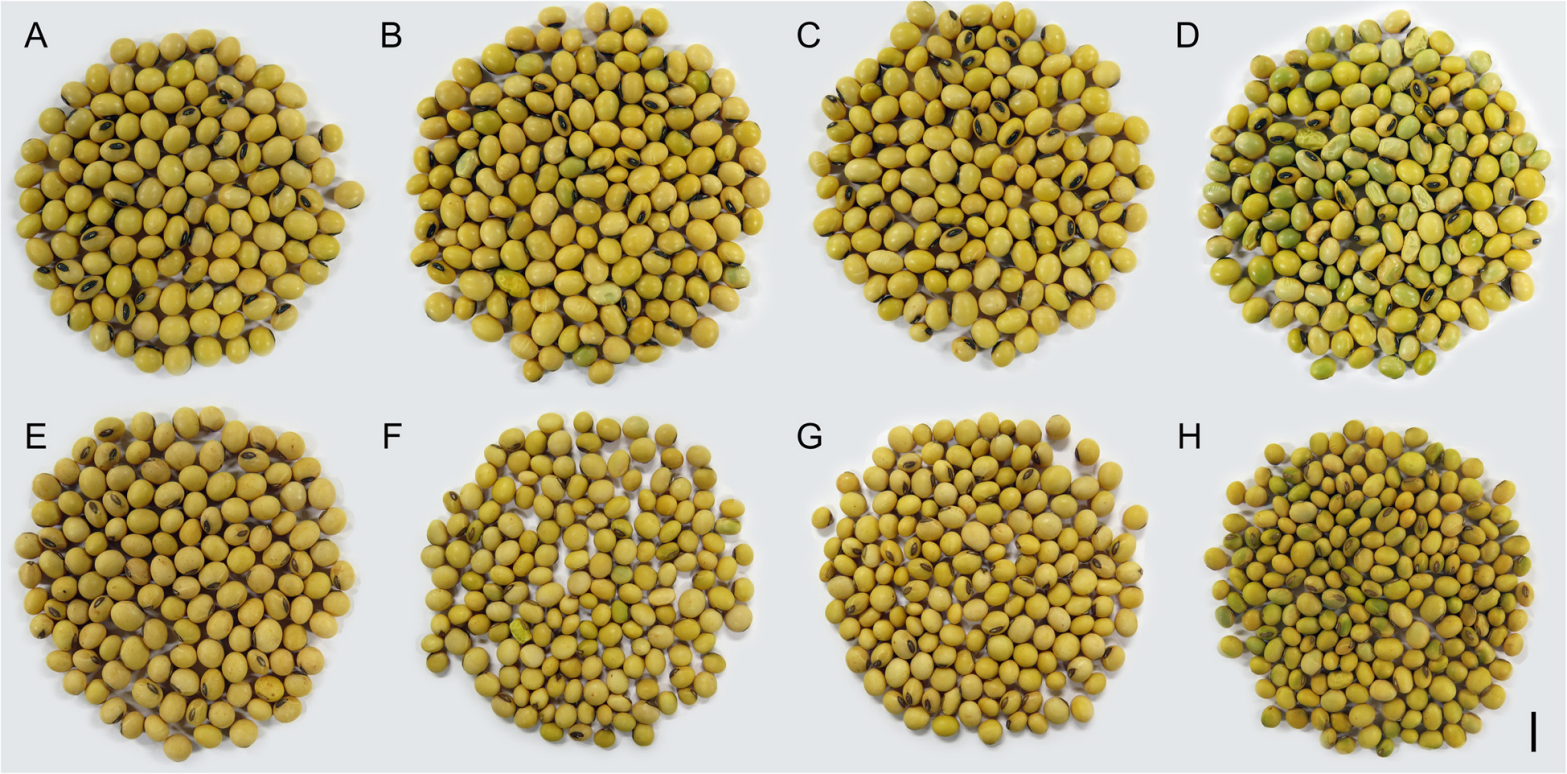
Non-stressed and stressed soybean seeds of the susceptible (top) and tolerant cultivar (bottom). **a** and **e**, non-stressed seeds; **b** and **f**, stressed seeds; **c** and **g**, yellow stressed seeds (selected from original stressed sample shown in **b** and **f**); **d** and **h**, green stressed seeds (selected from original stressed sample shown in **b** and **f**). Black bar in the right bottom corner represents 1 cm

**Fig. 3:**
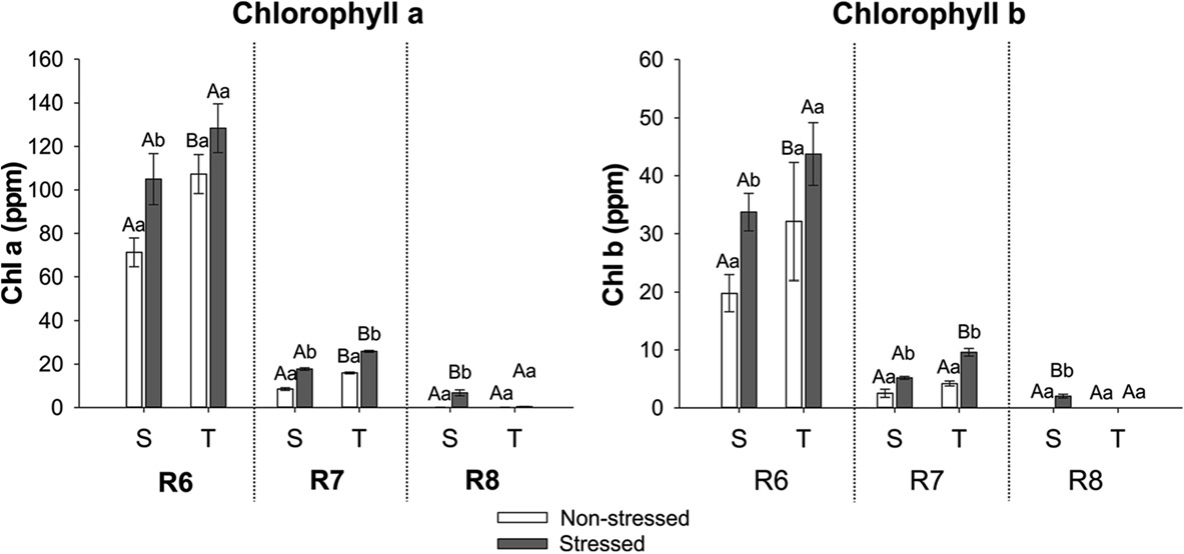
Chl *a* and *b* content of soybean seed samples produced under non-stressed (open bars) and stressed (closed bars) environmental condition by the susceptible (S) and tolerant (T) cultivars in three stages of maturation (R6-R7-R8). Bars represent the mean values ± SE of three independent biological replicates. Lowercase letters represent statistically significant differences (P ≤ 0.05) between different environmental conditions within the same cultivar and developmental stage and uppercase letters between different cultivars within the same environmental condition and developmental stage

Plants of susceptible and tolerant soybean cultivars were grown under non-stressed conditions and during the late seed maturation phase some plants were transferred to a condition of combined heat and drought stress. Seeds of both cultivars (matured under stressed and non-stressed conditions) were harvested in three stages of maturation (R6, R7 and R8) according to a scale proposed by SW Ritchie, JJ Hanway and HE Thompson [33] (Additional file 1: Table S1). R6 seed samples (stressed and non-stressed) were considered “green” if there was no sign of Chl degradation and they were still completely green. At R6 there were no appreciable differences in Chl degradation between the different cultivars and between environmental conditions (Fig. 1). At R7, when Chl degradation should have started, the greenish seeds were considered “green”. At this stage there was an expected decrease in percentage of green seeds due to normal chlorophyll degradation during maturation but no significant difference was observed between cultivars or environmental conditions (Fig. 1). At R8, when seeds should be yellow and the presence of Chl is a potential problem for seed quality, any sign of green pigmentation classified seeds as “green”, characterizing chlorophyll retention. At this stage, the susceptible cultivar produced, under stress, a significantly higher level of green seeds, reaching 22 % of green seeds against 1 % produced by the tolerant cultivar (Figs. 1 and 2). For more accuracy in determining the Chl retention in stressed soybean seeds, Chl *a* and Chl *b* were measured by HPLC (Fig. 3).

As expected, the chlorophyll content decreased during maturation of both cultivars, independently of the environmental condition. Chl *a* was present at higher levels in the seeds than Chl *b*. The effect of stress on seed chlorophyll content was variable depending on the cultivar and stage of maturation. At R8, when the presence of chlorophyll is a problem, there was only difference between stressed and non-stressed seeds of the susceptible cultivar and the levels of Chl *a* and *b* in stressed seeds of this cultivar were significantly higher than in stressed seeds of the tolerant material. This indicates that the difference in susceptibility to Chl retention between the two cultivars is genetic.

### Global gene expression profiling of stressed and non-stressed seeds of the susceptible soybean cultivar

A transcriptome analysis was carried out to identify changes in gene expression in soybean seeds of the susceptible cultivar, produced under non-stressed and combined heat and drought stress conditions during the late stages of maturation. The microarray used in this study covered 37,500 soybean transcripts. After normalization and background removal, data was filtered to keep probe sets with a signal above 3 in at least one of the conditions analysed, leaving 19,352 probe sets for further analysis.

R7 samples were composed of a mixture of seeds with variable levels of Chl degradation, which was considered to reflect slightly different stages of maturation. Based on these observations and on the results of PCA analysis (data not shown) we decided to use only data from the stages R6 and R8 for further analysis, in order to avoid ambiguous results.

**Fig. 4:**
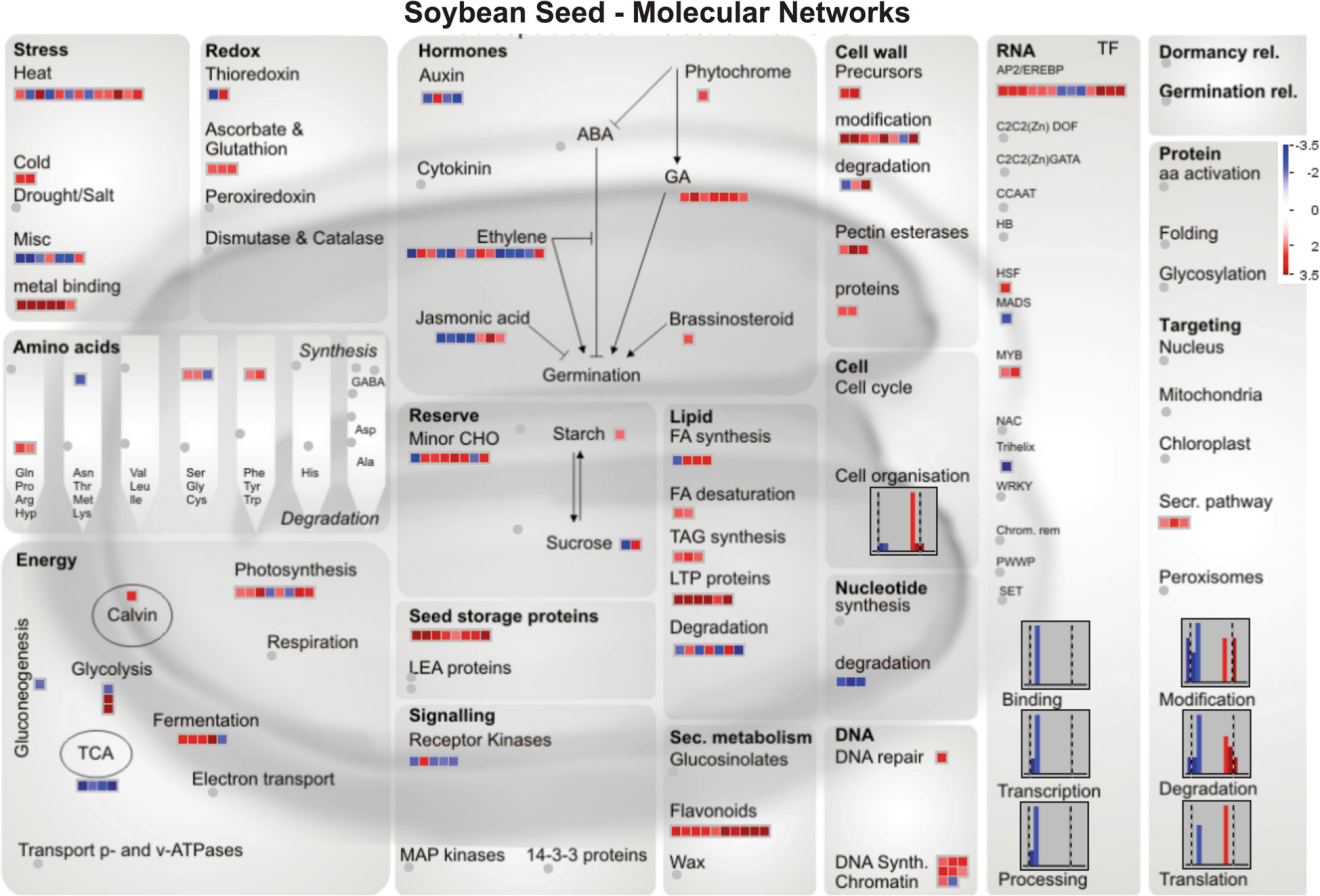
Seed MapMan molecular network map. Log2 ratios are used to express relative transcript levels in stressed versus non-stressed soybean seeds during maturation (R6-R8). Red squares depict higher levels in stressed seeds; blue squares higher levels in non-stressed seeds. Only ratios with P values lower or equal to 0.05 are displayed

For the differential expression analysis a comparison was made between stressed and non-stressed seeds during maturation, from R6 to R8. In total 833 genes exhibited at least a 2-fold change in expression (*p*-value ≤ 0.05 over three biological replicates). Expression of the selected genes was analysed with the seed-specific pathway of the Page-Man/MapMan package (http://MapMan.gabipd.org). This pathway efficiently captures the most relevant molecular processes in seeds (Fig. 4) [34] and it allows a global overview of the ontologies of the up- and down-regulated genes in stressed seeds, during the maturation process.

In the seed-specific pathways multiple ontologies were enriched. As was expected, the stress significantly changed expression of genes related to a large number of different cell functions. For example, photosynthesis, transcription factors such as AP2/EREBP, minor CHO (reserve), DNA repair/synthesis, cell wall modification, seed storage proteins, flavonoids (secondary metabolism), lipid transfer proteins and GA metabolism were among the up-regulated pathways. Genes related to TCA, nucleotide degradation and RNA binding, transcription and processing were down regulated as compared to non-stressed seeds.

We focused on genes related to photosynthesis and to genes encoding enzymes involved in the Chl degradation pathway, because our aim was to find genes related to Chl retention in soybean seeds. Expression of selected candidate genes were further studied using RT-qPCR analysis, comparing stressed and non-stressed seeds of the two soybean cultivars at R6 and R8 maturation stages.

### Expression analysis of Chl binding proteins in stressed and non-stressed soybean seeds

Chl degradation during leaf senescence is coupled to degradation of the Chl-protein complexes found in the thylakoid membrane, including PSI, PSII and cytochrome B_6_F [20, 24, 35]. Although we did not measure accumulation/degradation of the Chl-protein complexes, the expression of genes encoding for such proteins during the maturation process of stressed and non-stressed soybean seeds, can give us an idea of what happens with the photosystems and more specifically with the apoproteins that bind Chls.

**Fig. 5:**
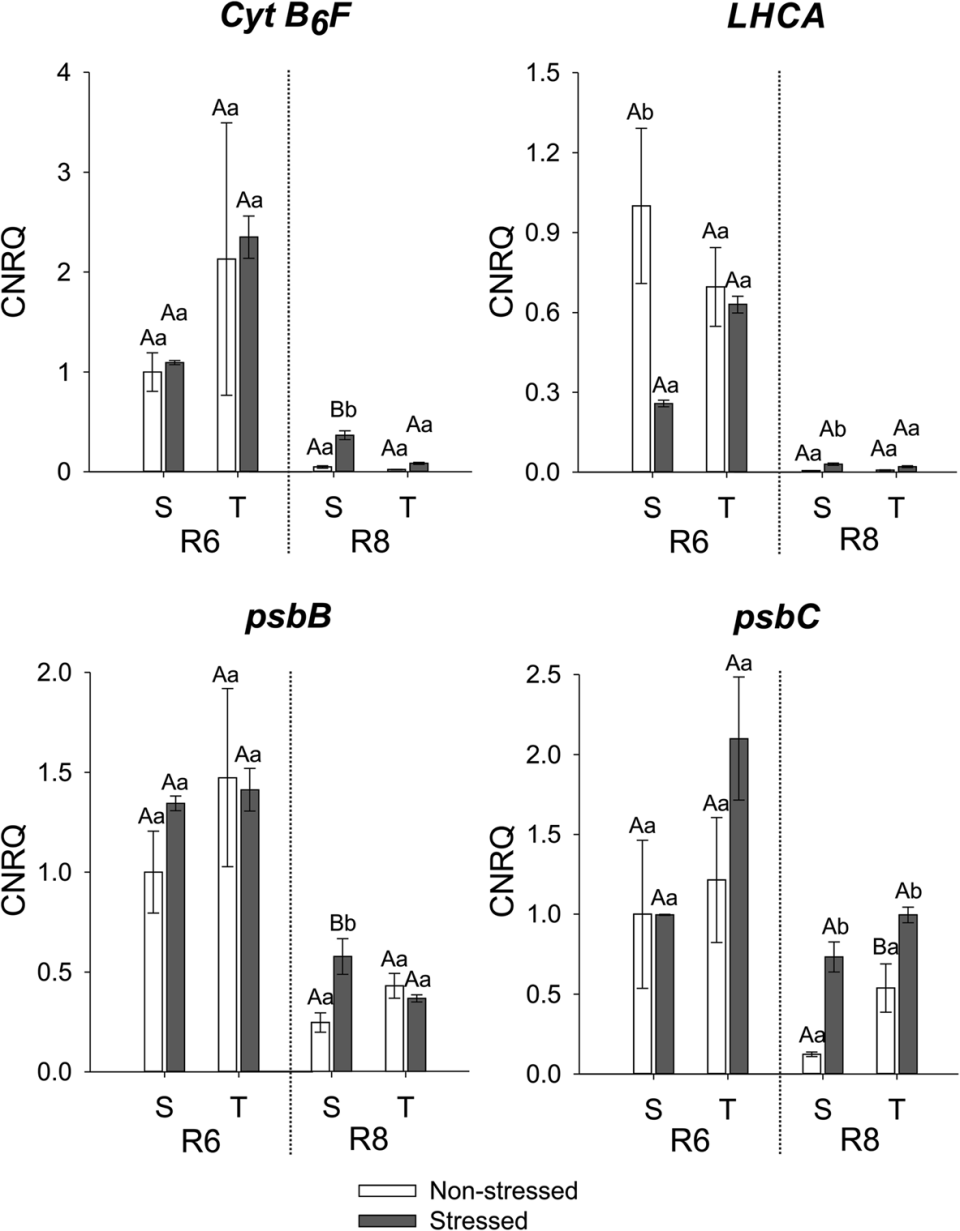
Gene expression analysis by real-time quantitative PCR represented as the calibrated normalized relative quantity (CNRQ) for *Cyt B6F*, *LHCA*, *psbB* and *psbC*. Gene expression is shown for non-stressed (open bars) and stressed seeds (closed bars) of the susceptible (S) and tolerant (T) cultivars in three stages of maturation (R6-R7-R8). Bars represent the mean values ± SE of three independent biological replicates. Lowercase letters represent statistically significant differences (P ≤ 0.05) between different environmental conditions within the same cultivar and developmental stage and uppercase letters between different cultivars within the same environmental condition and developmental stage

**Fig. 6:**
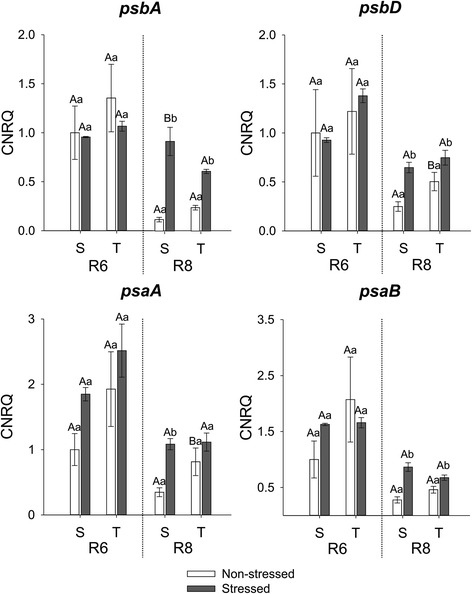
Gene expression analysis by real-time quantitative PCR represented as the calibrated normalized relative quantity (CNRQ) for *psbA*, *psbD*, *psaA* and *psaB*. Gene expression is shown for non-stressed (open bars) and stressed seeds (closed bars) of the susceptible (S) and tolerant (T) cultivars in three stages of maturation (R6-R7-R8). Bars represent the mean values ± SE of three independent biological replicates. Lowercase letters represent statistically significant differences (P ≤ 0.05) between different environmental conditions within the same cultivar and developmental stage and uppercase letters between different cultivars within the same environmental condition and developmental stage

The chosen transcripts represent some of the genes encoding for Chl-binding proteins, playing roles in photosystem I (*LHCA, psaA, psaB*), photosystem II (*psbA, psbB, psbC, psbD*) and cytochrome B_6_F (*Cyt B*_*6*_*F*). The expression of *Cyt B*_*6*_*F*, *LHCA* and *psbB* was higher in stressed than non-stressed seeds of the susceptible cultivar in R8 and there was significant difference in expression between stressed seeds of the susceptible cultivar and stressed seeds of the tolerant one. However, the expression of *psbC* was higher in stressed seeds of both cultivars in R8 (Fig. 5). *psbA* and *psbD* are genes encoding for the core proteins of PSII (D1 and D2 proteins, respectively). Expression of both genes towards the end of maturation (R8), was higher in stressed seeds of both cultivars. However, expression of *psbA* was significantly higher in stressed seeds of the susceptible cultivar compared to stressed seeds of the tolerant one while expression of *psbD* was higher in non-stressed seeds of the tolerant cultivar than in non-stressed seeds of the susceptible cultivar (Fig. 6). As the core subunits of PSI also bind Chl, the expression of the genes *psaA* and *psaB* encoding for the proteins A1 and A2 (respectively), was also analysed. At R8, expression of *psaA* was higher only in stressed seeds of the susceptible cultivar, while for *psbB* the expression was higher in stressed seeds of both cultivars (Fig. 6).

### Expression of CCGs in stressed and non-stressed soybean seeds

**Fig. 7:**
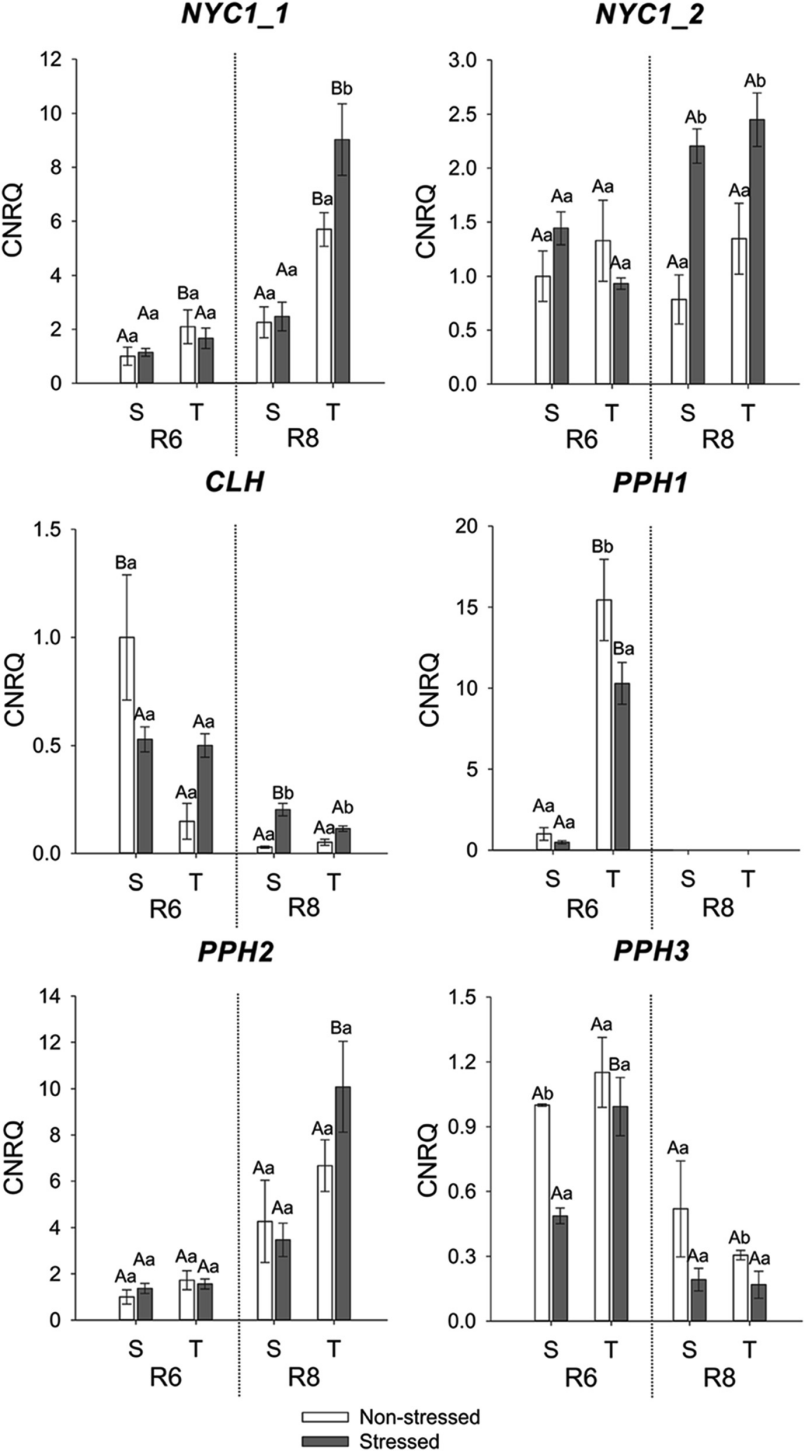
Gene expression analysis by real-time quantitative PCR represented as the calibrated normalized relative quantity (CNRQ) for *NYC1_1*, *NYC1_2*, *CLH, PPH1, PPH2* and *PPH3*. Gene expression is shown for non-stressed (open bars) and stressed seeds (closed bars) of the susceptible (S) and tolerant (T) cultivars in three stages of maturation (R6-R7-R8). Bars represent the mean values ± SE of three independent biological replicates. Lowercase letters represent statistically significant differences (P ≤ 0.05) between different environmental conditions within the same cultivar and developmental stage and uppercase letters between different cultivars within the same environmental condition and developmental stage

To better understand the breakdown of Chl in soybean seeds and, consequently, its retention, we analysed the expression of some of the genes encoding for enzymes in the Chl degradation pathway. Among the ten CCGs tested by RT-qPCR in the present study, *NYC1_1* and *PPH*2 were the most affected by stress and most likely to be related to Chl retention (Fig. 7).

The first step in Chl degradation is the conversion of Chl *b* to Chl *a* which is catalysed by Chl *b* reductase. Defects in the synthesis of this enzyme are known to cause Chl retention in leaves of rice *non-yellow colouring 1* (*nyc1*) mutants during senescence [20, 24]. The expression of two transcripts encoding for Chl *b* reductase (*NYC1_1* - Glyma07g09430 and *NYC1_2* - Glyma09g32370) was analysed. Although *NYC1_2* is higher expressed in stressed seeds of both cultivars in R8 (Fig. 7), Chl *b* is still present in stressed seeds of the susceptible cultivar at the end of the maturation (Fig. 3). However, expression of NYC1_1 is only higher in stressed seeds of the tolerant cultivar (Fig. 7), which could explain why mature seeds of this cultivar don’t contain Chl *b* (Fig. 3). This suggests that expression of both copies of *NYC1* might be necessary for complete conversion of Chl *b* to *a* in seeds of soybean.

Chl breakdown continues with the removal of Mg^2+^ and phytol by MCS and PPH/Chlorophyllases (CLH), resulting in Pheide *a*. The expression of four genes coding for CLH was analysed by RT-qPCR, but only one of them was expressed in the analysed samples (Glyma10g00570). The transcript level of this *CLH* gene was higher in stressed seeds of the susceptible cultivar in R8 both as compared to its non-stressed seeds as to the stressed seeds of the tolerant one. There was no difference between stressed and non-stressed seeds of the tolerant cultivar (Fig. 7). Despite these results, stressed mature seeds of the susceptible cultivar didn’t completely degrade the Chl (Figs. 2 and 3). As a result, no correlation was found between *CLH* expression and chlorophyll content (Fig. 10).

The mechanism of Mg-dechelation has not yet been fully resolved [10]. CLH, which hydrolyses phytol from Chl, was believed to be active during senescence-related Chl breakdown [36, 37]. However, so far *CLH* action has been classified as essential or not essential, depending on the species. The double mutant of AtCLH1 and AtCLH2 in Arabidopsis did not generate a stay-green phenotype [38] which suggested that *CLH* might not play a major role in Chl degradation during leaf senescence in Arabidopsis [20, 38]. Instead, the functional characterization of *PPH* has indicated that this enzyme is necessary for Chl breakdown in Arabidopsis [22] and rice [20].

The analysis of *PPH* expression in our seed samples indicates that pheophytinases may play a major role in dephytylation in seeds. The expression of *PPH1* (Glyma09g36010) and *PPH3* (Glyma12g01320) decreased during maturation, while *PPH2* (Glyma11g16070) increased (Fig. 7). *PPH2* expression seems to be required later in maturation, especially in stressed seeds of the tolerant cultivar. The expression of this gene was significantly higher in stressed seeds of the tolerant cultivar than in stressed seeds of the susceptible one. PPHs seem to be playing a major role in phytol removal in soybean seeds produced under stress, as stressed seeds of the tolerant cultivar do not retain greenness to the same extent as stressed seeds of the susceptible one (Fig. 7).

**Fig. 8:**
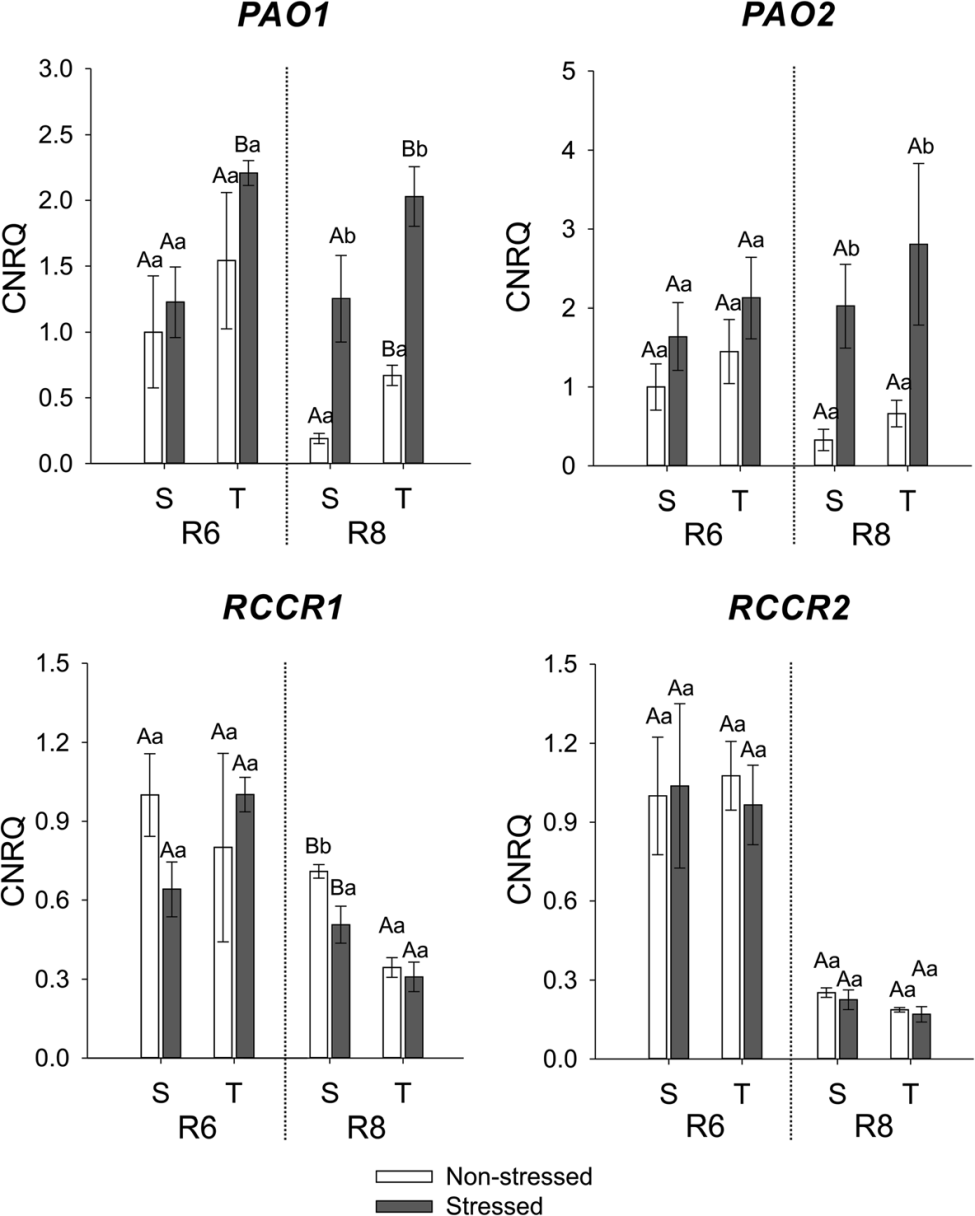
Gene expression analysis by real-time quantitative PCR represented as the calibrated normalized relative quantity (CNRQ) for *PAO1*, *PAO2*, *RCCR1* and *RCCR2*. Gene expression is shown for non-stressed (open bars) and stressed seeds (closed bars) of the susceptible (S) and tolerant (T) cultivars in three stages of maturation (R6-R7-R8). Bars represent the mean values ± SE of three independent biological replicates. Lowercase letters represent statistically significant differences (P ≤ 0.05) between different environmental conditions within the same cultivar and developmental stage and uppercase letters between different cultivars within the same environmental condition and developmental stage

Downstream of the degradation pathway, after Phein *a* is converted by PPH to Pheide *a*, this intermediate is subsequently converted to the red-coloured molecule RCC in a reaction mediated by PAO. The expression of two genes encoding for PAO was analysed: *PAO1* (Glyma11g19800) and *PAO2* (Glyma12g08740). Both genes were higher expressed in stressed seeds of the susceptible and tolerant cultivars mainly in R8, but the expression of *PAO1* was higher in both stressed and non-stressed seeds of the tolerant cultivar than in stressed and non-stressed seeds of the susceptible cultivar (Fig. 8). In the next step of Chl degradation, the red pigment (RCC) is converted to the non-coloured but blue-fluorescing product called primary fluorescent chlorophyll catabolite by RCCR. The primary fluorescent chlorophyll catabolite is further converted to non-fluorescent Chl-catabolites through different mechanisms depending on the species. The expression of *RCCR1* (Glyma14g01620) in R8 was lower in stressed than non-stressed seeds of the susceptible cultivar (Fig. 8). Also the level of expression was lower for both stressed and non-stressed seeds of the tolerant cultivar when compared with seeds of the susceptible one produced in R8. Independently of cultivar or stage of maturation, there was no difference in expression of *RCCR2* as result of the stresses applied (Fig. 8).

### Stay-green gene expression is impaired in stressed/green seeds

**Fig. 9:**
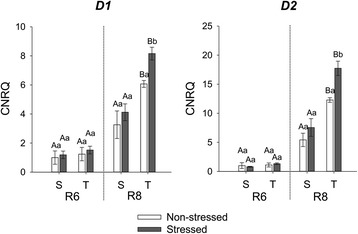
Gene expression analysis by real-time quantitative PCR represented as the calibrated normalized relative quantity (CNRQ) for *D1* and *D2*. Gene expression is shown for non-stressed (open bars) and stressed seeds (closed bars) of the susceptible (S) and tolerant (T) cultivars in three stages of maturation (R6-R7-R8). Bars represent the mean values ± SE of three independent biological replicates. Lowercase letters represent statistically significant differences (P ≤ 0.05) between different environmental conditions within the same cultivar and developmental stage and uppercase letters between different cultivars within the same environmental condition and developmental stage

The expression of both *D1* (Glyma01g42390) and *D2* (Glyma11g02980) in soybean seeds increased during the progression of seed maturation, with higher levels in R8 (Fig. 9). In R8 the expression of both genes was higher in stressed than non-stressed seeds only of the tolerant cultivar which exhibited consistently higher expression levels of both genes when compared with the susceptible cultivar.

**Fig. 10:**
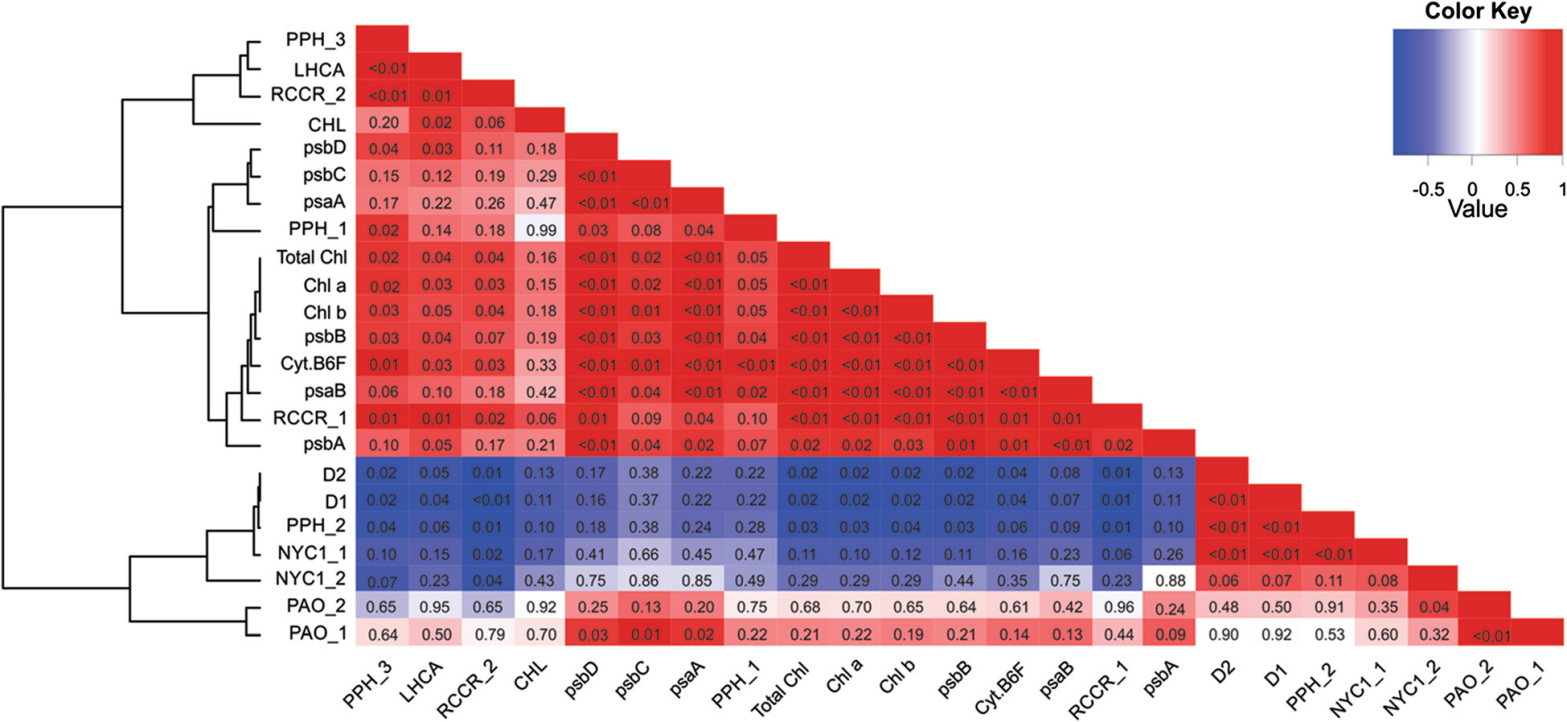
Heat map of correlations. Each square represents the Spearman correlation coefficient between expression of genes related to chlorophyll degradation and chlorophyll content. Traits were classified by hierarchical clustering using the distance function 1-correlation in R. The significance of the correlations were calculated as ANOVA p-values and are represented by asterisks (** *P* < 0.01 and * *P* < 0.05)

In general, most of the genes analysed follow a pattern of decreased expression together with a decrease in chlorophyll content during seed maturation, except for *NYC1_1* and *2*, *PPH2*, *D1* and *D2*. In the correlation analysis (Fig. 10) it is evident that only expression of *D1*, *D2*, *NYC1_1* and *PPH2* showed a significant negative correlation with chlorophyll content, while all the other genes were either positively correlated or showed no correlation with chlorophyll content.

## Discussion

For over a decade Brazilian soybean growers have been reporting the occurrence of green seeds at the end of the maturation process and it has become a great concern to the soybean market in the last few years ([39,40]). However, chlorophyll retention in seeds is not unique to Brazil or to soybean as it has been reported in other countries and in canola seeds [2–7, 37, 41–43]. A similar phenotype to chlorophyll retention, called stay-green has been described in leaves and seeds of mutants of different species [44]. Although several of these mutations have been described for the actual *SGR* gene [19, 21, 45] there are also mutations described in CCGs resulting in a stay-green phenotype, for example: *PAO* [46, 47], *OsNYC1* [35], *AtPPH* [22], and *OsNYC3* [28].

Although reports of Chl retention in seeds can be tracked to the early nineties [6, 41], the molecular basis of this phenomenon has not yet been unravelled. Using a RT-qPCR approach we demonstrate that combined heat and drought stress affect expression of genes in the Chl degradation pathway, as well as genes encoding Chl-binding proteins in at least one cultivar and/or stage of maturation. Under these stressful environmental conditions the two soybean cultivars also behaved differently regarding the chlorophyll retention phenotype. Based on our results we suggest that impairment of expression of *D1*, *D2*, *NYC1_1* and *PPH2*, is the (downstream) cause of chlorophyll retention in mature soybean seeds of the susceptible cultivar.

### How can impaired expression of SGR and CCGs lead to Chl retention in stressed soybean seeds

During soybean seed maturation, *D1* and *D2* displayed very similar patterns of expression (Fig. 9) as has been observed also during leaf senescence [17]. D1 and D2 are two unlinked nuclear loci that have been assumed to be paralogues in tetraploid soybean because homozygosity at both nuclear loci is required for the stay green phenotype, unlike other species in which the mutation of one gene has been shown to be sufficient for the stay-green phenotype [17].

The increase in expression of both genes correlated with chlorophyll degradation during seed maturation and the higher expression of *D1* and *D2* in stressed seeds of the tolerant cultivar at R8 supported the more efficient Chl degradation of this cultivar even under stressed conditions. However, seeds of the tolerant cultivar also expressed higher levels of both stay-green genes under non-stressed conditions when compared to the susceptible cultivar. This constitutive difference in levels of expression of *SGR* between the two cultivars may explain the difference in the efficiency of disassembly of the Chl-protein complexes and Chl degradation between the two cultivars under heat and drought stress. Apparently, under normal environmental conditions the higher levels of SGR transcripts in seeds of the susceptible cultivar do not cause phenotypic differences but under stressed environmental conditions it clearly leads to a Chl retention phenotype suggesting cryptic genetic variation between these two genotypes [48, 49].

Besides the importance of SGR in the activation of Chl-protein complex disassembly and, hence, Chl-degradation in leaves [19, 21], it has been shown that the *SGR1* and *SGR2* genes are also necessary for seed degreening in Arabidopsis [50]. Interestingly the soybean *D1* and *D2* genes, which are functional orthologues of SGR in other species, promoted restoration of the wild type phenotype when introduced into the Arabidopsis SGR mutant *nye1* [17]. Furthermore, it was recently found that the Arabidopsis SGR1 physically interacts with all six known CCEs (i.e. NOL, NYC1, HCAR, PPH, PAO, and RCCR) and possibly forms a highly dynamic, multi-protein complex for Chl degradation during natural senescence [11, 51].

The transcription of some Chl-degradation genes, especially *NYC1* and *PPH*, were shown to be affected by the *d1d2* mutation in a previous study with soybean [17]. Consistent with this, the expression of *NYC1_1* and *PPH2*, mirrored the expression of *D1* and *D2* in soybean seeds. In fact, *D1* and *D2* are highly correlated with expression of *NYC1_1* and *PPH2*, suggesting co-regulation of these genes (Fig. 10). *D1, D2, PPH2* and *NYC1_1* are the only genes with a significant negative correlation with chlorophyll content (with p-values 0.02, 0.02, 0.03 and 0.1 respectively). Therefore we hypothesise that the constitutively higher expression of these four genes aid greater tolerance to heat and drought stress regarding chlorophyll degradation in the seeds during maturation. Future experiments overexpressing these genes in the susceptible cultivar will be needed to test this hypothesis.

Approximately 75 % of the genes are present in more than one copy in the soybean genome [52] due to at least two whole genome duplication (WGD) events within the last 60 million years [53–55]. However, it is hypothesised that functions of duplicated genes can be very divergent [17]. This divergence is well exemplified by the terminal flower gene. Among the four homologous genes of Arabidopsis TERMINAL FLOWER (AtTFL1) in soybean, only one has been found to control growth habit whereas the other copies are suggested to have alternative functions as they show divergent transcriptional patterns [56]. This also seems to be the case for the genes involved in seed degreening, especially *NYC1_1* and *PPH2*, which are the only copies that where strongly affected in stressed seeds.

The Chl-binding proteins, on the other hand, correlated positively with the chlorophyll content of the seeds. The disassembly of the photosystems has been consistently reported to be directly related to Chl degradation during senescence. For example, the breakdown of the LHCs, is crucial for degreening during leaf senescence [18, 57], as these peripheral antennas contain most of the Chl. Consequently, retention of LHCs and of core subunits of the PSII have been associated with the stay-green phenotype in some species [10, 11, 17, 35, 58–60]. The higher expression of *psbC* and *psbB* observed in mature stressed/green seeds was also reported in the rice stay-green mutant *nyc4-1* in which the PSII subunits CP43 and CP47 (encoded by *psbC* and *psbB* respectively) were more stable than in the wild type during senescence [24].

Under stressful conditions the changes in expression of *psbA* and *psbD* were different in seeds of the susceptible and tolerant cultivar. However, a possible retention of D1 protein (*psbA*) requires further investigation since this protein has the highest turnover among all the PSII proteins and its abundance can be affected by several processes [61, 62].

Although there are no reports so far about photosystem disassembly in seeds, we expect that the degreening in seeds also depends on the disassembly of LHCs and of other Chl-binding proteins. With the exception of *psbA*, which shows constant expression during maturation in stressed seeds of the susceptible cultivar, the pattern of expression of *LHCA*, *Cyt B*_*6*_*F* and core proteins of PSI and PSII decreased in both non-stressed and stressed seeds during maturation. This mostly reflects the normal disassembly of the photosynthetic apparatus during the maturation process that follows Chl degradation.

The role of SGR genes as regulators of chlorophyll degradation during developmental and dark-induced senescence is well established in leaves and more recently the role of these genes in embryo degreening has been described [50]. During Arabidopsis leaf senescence *SGR1* and *SGR2* act antagonistically in the control of Chl degradation [63], while in seeds they act synergistically [17, 50]. Although there has been great progress in understanding chlorophyll degradation in higher plants over the last few years, the seed degreening process still awaits to be resolved. Studying *in vitro* interactions between SGR, Chl-binding proteins and CCEs may possibly elucidate some aspects of Chl degradation during seed ripening.

## Conclusion

The combination of heat and drought stress during late seed maturation resulted in a “mild” stay-green phenotype and impaired expression of *SGR 1* and *2* (*D1*, *D2*), *PPH2* and *NYC1_1* in soybean seeds of a susceptible soybean cultivar. This may be a case of cryptic genetic variation between these two genotypes as the genetic variation itself does not lead to chlorophyll retention under normal environmental conditions, but under combined heat and drought stress a clear green phenotype is observed in seeds of the susceptible cultivar. In other words we suggest that the constitutively higher expression of these four genes in fully mature seeds of the tolerant cultivar allows these seeds to cope with stressful conditions and complete chlorophyll degradation. The results described here deepen our understanding of chlorophyll retention in soybean seeds. The insights gained from this research will pave the way for new research ultimately providing direction to breeding programs aiming to produce tolerant cultivars. This is particularly pertinent as the hot and dry climate conditions which cause chlorophyll retention are increasingly common in the largest soybean production areas of the world and as a result there is an increasing need for resistant germplasm.

## Methods

### Plant material and growth conditions

Two cultivars of soybean were used to produce the seeds in this study. The cultivar MG/BR 46-Conquista (BRA093483/CENARGEN) (late cycle) was used as susceptible to Chl retention and the cultivar BRS 133 (BRA097934/CENARGEN) (semi-early cycle) was used as the tolerant genotype [9].

During the vegetative phase both cultivars were grown in a polytunnel, subjected to the local environmental conditions (Botucatu, SP – Brazil, 2013) (Additional file 1: Figure S1 and Figure S2). During this phase the climatic conditions of this region could be considered as non-stressed with an average temperature of 24.7 °C. The plants received a free water supplement (field capacity). At R5.5 (Additional file 1: Figure S3) part of the pots were maintained under these non-stressed conditions and part was transferred to a greenhouse (Van der Hoeven, Brazil) with an average day temperature of 39.5 °C for the susceptible cultivar and 38.7 °C for the tolerant one (Additional file 1: Figure S1 and Figure S2). The plants in stressed conditions were only watered when wilting was observed. At these moments plants were watered to bring the soil to field capacity.

### Sampling points

The seeds were harvested at three stages of maturation (R6, R7 and R8) solely based on plant and pod characteristics (Additional file 1: Table S1). At each stage the four plants in the pot (six pots) were cut and the pods were removed. A post-harvest selection based on seed colour (Additional file 1: Figure S4) was done in order to reduce variability in maturation between seeds of the same sample.

Combining the environmental conditions (non-stressed and stressed) and the harvesting points (R6-R7-R8), there were six different seed samples for each cultivar and six replicates. The six replicates of each sample were grouped two by two to constitute three biological replicates. The appearance of the pods and seeds constituting non-stressed samples in each of the three stages of maturation is displayed in Figure S4 (Additional file 1).

### Chl quantification

Samples were lyophilized and ground to a powder for the quantification of Chl *a* and *b* by high performance liquid chromatography (HPLC). Both Chl *a* and *b* standards were purchased from Sigma (USA). All the solvents used were HPLC grade.

Extraction of Chls was performed under green light according to SS Teng and BH Chen [64] with adaptations. Ground seed samples of 0.5 to 2 g were placed in a 50 ml Falcon tube, to which 5 ml of methanol was added. The mixture was homogenized with a homogenizer for 30s and centrifuged at 3000 g (4 °C) for 3 min. The supernatant was collected in a new Falcon tube. In a second step the same procedure was repeated with acetone, and this was repeated several times until the extract became colourless. All the methanol/acetone extracts were pooled and brought to a volume of 25 or 50 ml with the addition of methanol and the solution was filtered through a 0.2 μm membrane filter for HPLC and placed in an amber vial for immediate subsequent injection. The extraction of Chls was done in a ratio of 1:100 (w/v) for R6 samples (intense green), 1:50 (w/v) for R7 samples (light green) and 1:12.5 (w/v) for R8 samples depending on the intensity of the pigment colour (Chl concentration).

Analytical separations were performed on a Shimadzu Shim-Pack VP-ODS column (150 x 2.0 mm - serial number 6102708). Pigments were eluted using a linear gradient in 15 min, with a two-solvent system: methanol:ammonium acetate (80:20 v/v) and methanol:acetone (80:20 v/v). The injection volume was 25 μl. The fluorescence detector (Shimadzu RF-10XL) was operated at an excitation of 440 nm, an emission of 660 nm and a flow rate of 0.8 ml/min [65].

Calibration curves were made from 0.01 to 5.0 mg/l for both Chls. High linearity was obtained with correlation coefficients of 0.99. Chl *a* and *b* were then quantified using the respective calibration curves.

### RNA extraction and cDNA synthesis

Total RNA extraction was performed with the NucleoSpin® RNA Plant kit (Macherey-Nagel) according to the manufacture’s protocol in three biological replicates with at least 15 seeds of each biological sample. RNA integrity was assessed by analysis on a 1 % agarose gel and RNA sample quality and concentration were additionally assessed using a Nanodrop ND-1000 (Thermo Scientific). cDNA was then synthesized using the High-Capacity cDNA Reverse Transcription Kit (Applied Biosystems) according to the manufacture’s protocol.

### Microarray analysis

For the microarray analysis, three biological replicates of total RNA from seed samples of the susceptible cultivar (MG/BR 46) were used. After cDNA synthesis, according to the Affymetrix protocol (Affymetrix®, GeneChip® 3’IVT Express Kit user manual), the samples were hybridized to Affymetrix microarrays (Soybean Genome GeneChip Array). In total 18 arrays were used (3 stages of maturation X 2 environmental conditions X 3 replicates, GEO Series ID GSE70999).

Quality control and pre-processing analysis of the microarray results was done using a tool of the open source package ArrayAnalysis.org that is freely available from http://www.arrayanalysis.org [66]. After normalization and background removal, data was filtered to keep probe sets with a signal above expression value 3 in at least one of the conditions analysed.

To get a shorter list of candidate genes related to stress and thus Chl retention in soybean seeds we used R. Data was corrected for multiple testing (FDR). A 2-fold change and significant level of 0.05 (*p*-value ≤ 0.05) were used for filtering.

### Quantitative PCR (RT-qPCR) analysis

iQ-SYBR Green-Supermix (Bio-Rad) was used for gene expression analysis on a MyIQ RT-qPCR machine (Bio-Rad) and Lumino Ct SYBR Green RT-qPCR Ready Mix (Sigma) for the analysis on an Eco Real-Time PCR system (Illumina).

The RT-qPCR program for both machines consisted of a first step at 95 °C for 3 min, followed by 40 cycles of 15 s at 95 °C and 1 min at 60 °C. The primers used (Additional file 1: Tables S2 and Table S3) were preferably designed in the 3’ end of the transcript and, if possible, spanning an intron–exon border. The Tm of the primers was between 58 and 62 °C except a few primers with slightly lower Tm. Routinely a melting curve analysis was performed after the RT-qPCR run (between 55 and 95 °C with 0.5 °C increments each 10 s). For all primers, a single peak was observed, confirming the synthesis of a single product.

Primer efficiency was calculated performing a 10X dilution series and the slope of the standard curve was translated into an efficiency value. All primers showed efficiencies between 90 and 110 %. Ct values were analysed in an excel sheet using a normalization factor calculation based on the geometric mean of multiple control genes proposed by J Vandesompele, Kd Pretter, F Pattyn, B Poppe, NV Roy, Ad Paepe and F Speleman [67]. RT-qPCR data of each gene of interest were normalized against the two most stable reference genes, chosen as described below. The list of target genes analysed in this study can be found in Additional file 1: Table S2.

### Reference gene identification

The identification of new reference genes, suitable for normalization of RT-qPCR data, was done based on microarray data. The most stable transcripts were selected based on the datasets from three microarray experiments: this study, KA Hudson [68] and T Asakura, T Tamura, K Terauchi, T Narikawa, K Yagasaki, Y Ishimaru and K Abe [69] (GEO Series ID GSE70999, GSE18827 and GSE26443, respectively).

The expression of the selected genes was analysed by RT-qPCR and their stability was calculated by geNORM [67]. Two genes (Prot and 60S) displayed M values lower than 0.5 (Additional file 1: Figure S5) and were used for RT-qPCR expression data normalization in this study.

### Statistical analysis

An ANOVA test was performed on the percentage of green seeds, Chl content and gene expression data at the significance level of 5 % (*p* ≤ 0.05). A factorial 2 X 2 set-up (2 cultivars X 2 environmental conditions) was used and the analysis was done for each stage of maturation individually. The averages were compared by T test at 5 % probability level.

### Correlation analysis

Correlation analysis was performed between the expression of 20 genes and chlorophyll content in the seeds. The heat map was constructed using the Spearman correlation coefficient matrices R-packages “MASS”, “Hmisc” and “VGAM” and the R graphic packages “gplots” and “graphics” were used for visualizing the data.

## Availability of supporting data

The data set supporting the results of this article is available in the NCBI’s GEO dataset (http://www.ncbi.nlm.nih.gov/gds) under the series ID GSE70999.

## Additional file

Additional file 1: Table S1. Description of the vegetative and reproductive stages of soybean (RITCHIE et al., 1982). **Table S2**. Sequences of the primers used for the target gene study. **Table S3**. Sequences of the primers used for the analysis of reference genes. **Figure S1**. Average temperatures and relative humidity during the life cycle of the susceptible cultivar under non-stressed and stressed conditions. **Figure S2**. Average temperatures and relative humidity during the life cycle of the tolerant cultivar under non-stressed and stressed conditions. **Figure S3**. Progression of seed development in the stage of maturation R5 (R5.3; R5.4; R5.5), cultivar MG/BR 46. The black bar represents 1 cm. **Figure S4**. Pod and seed characteristics at the reproductive stages R6, R7 and R8, cultivar MG/BR 46. The black bar represents 1 cm. (PDF 1714 kb)

## Abbreviations

CCE: chlorophyll catabolic enzyme
CCG: chlorophyll catabolic gene
Chl: chlorophyll
CLH: chlorophyllase
HCAR: hydroxyl-methyl chlorophyll a reductase
HPLC: high performance liquid chromatography
LHC: light harvesting complex
NOL: non-yellow coloring like
NYC: non-yellow coloring
PAO: pheophorbide *a* oxigenase
Pheide: pheophorbide
Phein: pheophytin
PPH: pheophytinase
PSI: photosystem I
PSII: photosystem II
RCC: red chlorophyll catabolite
RCCR: red chlorophyll catabolite reductase
SGR: stay-green
