# Supplementary material for: Gene expression profiling of the green seed problem in Soybean
Source: BMC Plant Biol. 2016 Feb 1;16:37. doi: 10.1186/s12870-016-0729-0 (PMC4736698; doi:10.1186/s12870-016-0729-0)
Supplement: Additional file 1: Table S1. — Description of the vegetative and reproductive stages of soybean (RITCHIE et al., 1982). Table S2. Sequences of the primers used for the target gene study. Table S3. Sequences of the primers used for the analysis of reference genes. Figure S1. Average temperatures and relative humidity during the life cycle of the susceptible cultivar under non-stressed and stressed conditions. Figure S2. Average temperatures and relative humidity during the life cycle of the tolerant cultivar under non-stressed and stressed conditions. Figure S3. Progression of seed development in the stage of maturation R5 (R5.3; R5.4; R5.5), cultivar MG/BR 46. The black bar represents 1 cm. Figure S4. Pod and seed characteristics at the reproductive stages R6, R7 and R8, cultivar MG/BR 46. The black bar represents 1 cm. (PDF 1714 kb) [file 12870_2016_729_MOESM1_ESM.pdf]

Supplementary Data

The plants (4 plants/pot) were grown in pots of 15l filled with sandy loam soil. The soil was fertilized and limed according to the crop requirements. The analysis of the phenological stages of the plants were based on the scale proposed by Ritchie et al. (1982) (Table S1) with slight modifications, based on seed morphological characteristics, in order to better characterize the seed maturation (Figure S4).

Table S1. Description of the vegetative and reproductive stages of soybean (RITCHIE et al., 1982).

Stage of Maturation	Nomination	Description
VE	Emergence	Cotyledons above the soil surface
VC	Cotyledon	Cotyledons fully unfolded
V1	First node	Unifoliate leaf fully unfolded
V2	Second node	First trifoliate leaf fully unfolded
V3	Third node	Second trifoliate leaf fully unfolded
Vn	nth node	Ante-nth trifoliate leaf fully unfolded
R1	Beginning bloom	One open flower at any node on the main stem
R2	Full bloom	Open flower at one of the two uppermost nodes on the main stem with a fully developed leaf
R3	Beginning pod	Pod is 5mm long at one of the four uppermost nodes on the main stem with a fully developed leaf
R4	Full pod	Pod is 2cm long at one of the four uppermost nodes on the main stem with a fully developed leaf
R5	Beginning seed	Seed is 3 mm long in the pod at the for uppermost nodes of the main stem with a fully developed leaf
R5.1	~10% filling	Seed is ~5mm long in the pod (perceptible to the touch)
R5.2	~11-25% filling	Seed is ~7mm long in the pod
R5.3	~26-50% filling	Seed is ~8mm long in the pod
R5.4	~51-75% filling	Seed is ~10mm long in the pod
R5.5	~76-99% filling	Seed is ~11mm long in the pod
R6	Full seed	Pod containing a green seed that fills the pod cavity at one of the four uppermost nodes on the main stem with a fully developed leaf (plant with green leaves)
R7	Begin of maturation	One normal pod on the main stem that has reached its mature pod color
R8	Full maturity	Ninety-five percent of the pods have reached their mature pod color

Table S2. Sequences of the primers used for the target gene study.

Gene		Forward	Reverse	Reference
NYC1_1	Glyma07g09430	TCGGGAGTTTCTTCTTTCTGGA	ACAAACATCACAAGCAATGCCTAC A	Fang et al. (2014)
NYC1_2	Glyma09g32370	GCAACAACAAGCCATCTCAAA	GGCCATGGATGTGATGATAGAA	This study
PAO_1	Glyma11g19800	CTTGTGTTAAGATTCCTCAGGCTT	ACCATTCTCATCAGGCCATACA	Fang et al. (2014)
PAO_2	Glyma12g08740	CAACCATTGCCATCAACTGTT	TTGCACAAAACACAACTGTTGTA	Fang et al. (2014)
RCCR_1	Glyma14g01620	GACCCACAACAACCAA	TGCAAGGAAGAAGCTGAGT	This study
RCCR_2	Glyma02g47120	CTCTCTGTTCCATCTTCTTCTTTG	GAAACGAGATCAACCATGAGG	Fang et al. (2014)
PPH_1	Glyma09g36010	TGGTGGAGTTTATTATACCAA	CCAATCACATAGCCATCGC	Fang et al. (2014)
PPH_2	Glyma11g16070	GCTGCCAAGTTGTGAACTCA	AATCTCACAGGCGCACATGC	Fang et al. (2014)
PPH_3	Glyma12g01320	ATGCTGTCAAGCAGTGGATAT	TCTCTTTGCAGCTCCTTATTG	Fang et al. (2014)
D1	Glyma01g42390	CAAGGAAGAGAGTGAGCAAGA	CACTGAATTGGGCTTAACGTC	Fang et al. (2014)
D2	Glyma11g02980	CACTCCTAAGAACAAAACCTTCAG T	TTTTCCCTACTGTGGAAGACGG	Fang et al. (2014)
CHL	Glyma10g00570	ACACCTCTTGTCTACGTCC	ACTCCTCCAACAAACCTTCTC	This study
LHCA	Glyma16g26130	ACCCATGGCACAACAACA	ACAGCACAGCGATACCAAC	This study
Cyt B6F	Glyma12g32580	CGTCCCTCTGTTGTCATGT	GGAGAGGTGATGGTGAAAAGTT	This study
psaA	---	AGCAACTCCCTTTTTCACC	GACCCGCTATCAAGAAAAGAAT	This study
psaB	---	TGGTGTTTATCAGTGGTGGT	TGATGATTGAGGCGGGATT	This study
psbA	Glyma13g15560	GCAAACCTATAGCCGCAGA	GGATGGTTTGGTGTTTTGATGA	This study
psbB	---	CCCTCTGACCCTGTTCTT	ATATTCCAACCGCCCCAC	This study
psbC	---	CCTAGTAGTTTGCCGGAT	CACGTGGAAACGCTCTTTA	This study
psbD	---	AACGAAGTCATAGGCACG	CTTTGGGGTTGCTTTTTCC	This study

Table S3. Sequences of the primers used for the analysis of reference genes.

Primer set	Annotation	Gene	Forward	Reverse	Frag. Length
UBQ	Ubiquitin	Glyma06g20310	CGAAGAGTGAGGGAAATTGT	GCCATCACTGCAACCAAA	102
SMP	Seed Maturation Protein	Glyma20g37670	ACAAGTCCTTCACCCCAAT	TAATCCTCCAAGCCCCACC	196
Aqua	Aquaporin	Glyma14g06680	TTTGTTGCCCTCTTCCCCC	CCCCACTTCTCACTCTCACC	114
Asp Aminoprot	Aspartate Aminotransferase	Glyma06g08670	TCTCCAACCTCCGCTTCT	GTTCAACTCGCCTCACTACA	184
Prot	Proteasome (20S subunit beta)	Glyma06g08260	CACCAACACACGATACAACT	TCCCAACCACCAACAATTAACC	122
60S	60S	Glyma15g42620	AGGCAGAGAAGGAGGAGAA	AAAGAAAACCTAGCACCCAAG	133

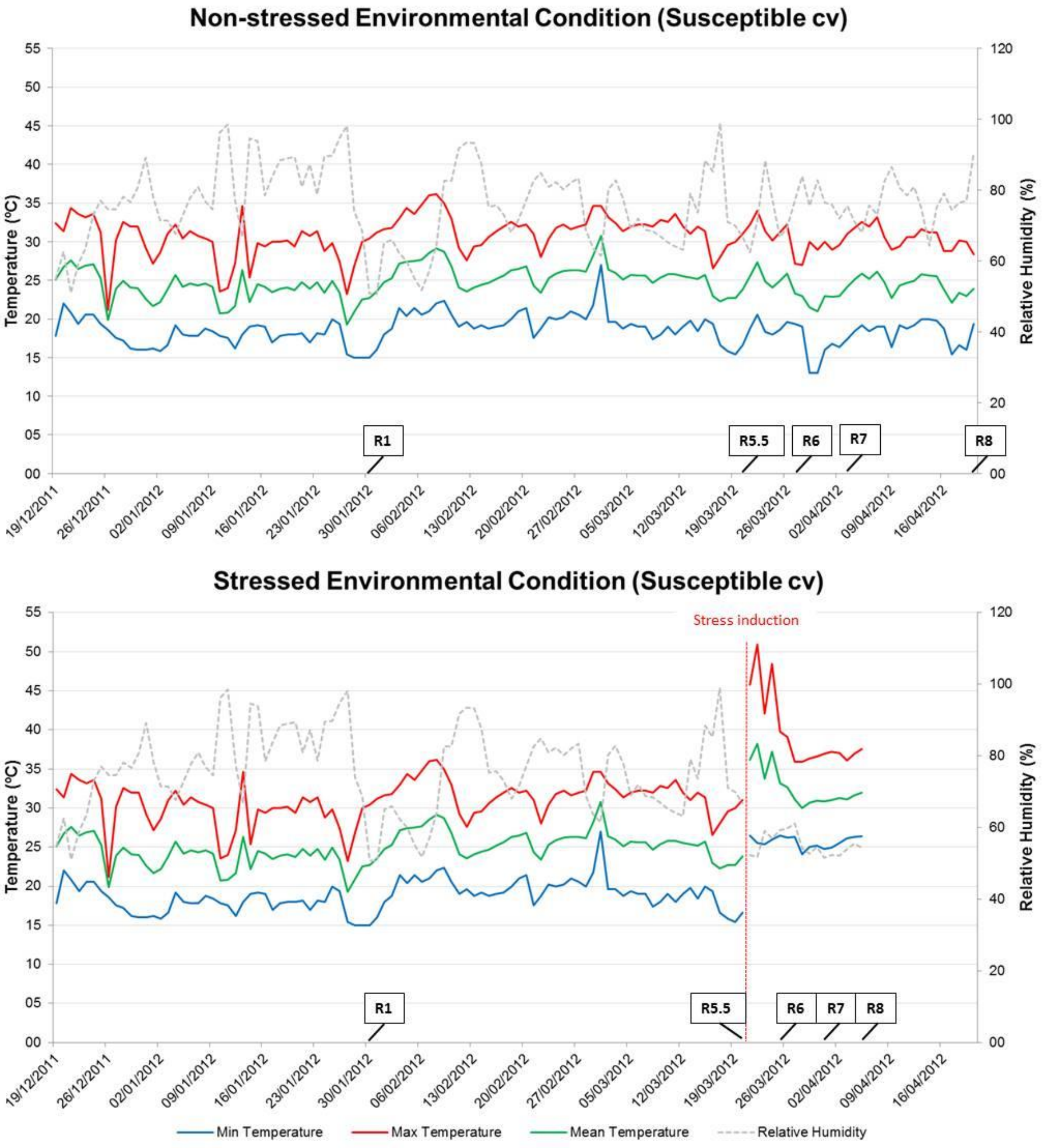

Figure. S1. Average temperatures and relative humidity during the life cycle of the susceptible cultivar under non-stressed and stressed conditions.

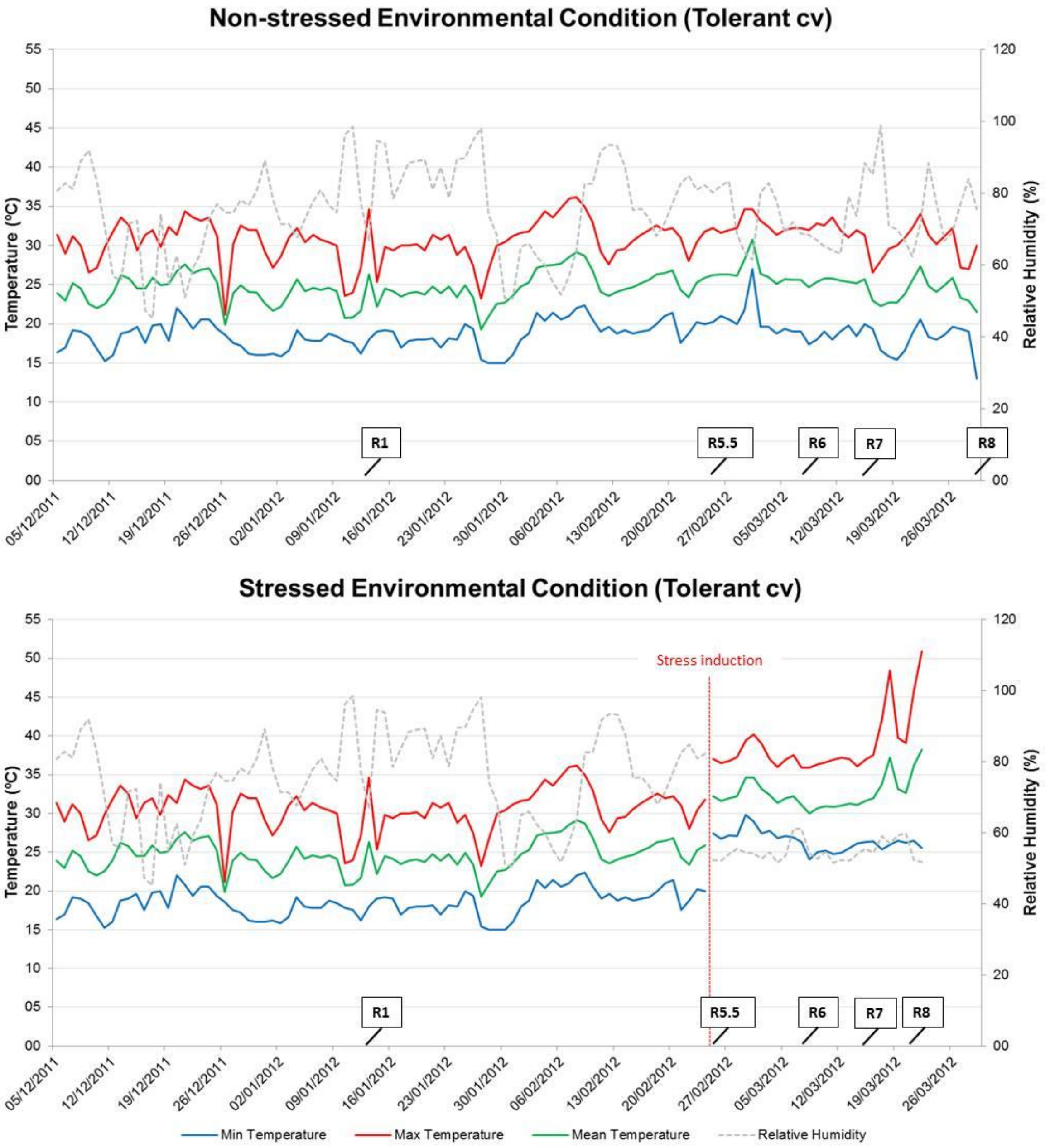

Figure S2. Average temperatures and relative humidity during the life cycle of the tolerant cultivar under non-stressed and stressed conditions.

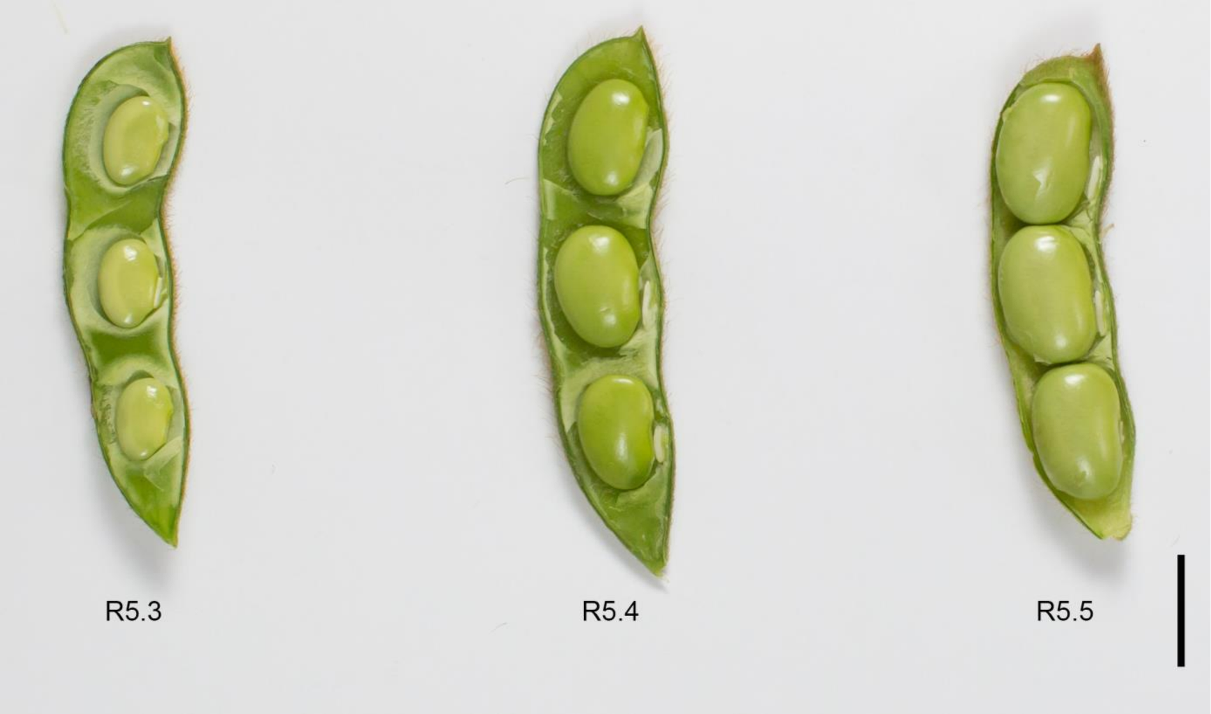

Figure S3. Progression of seed development in the stage of maturation R5 (R5.3; R5.4; R5.5), cultivar MG/BR 46. The black bar represents 1cm.

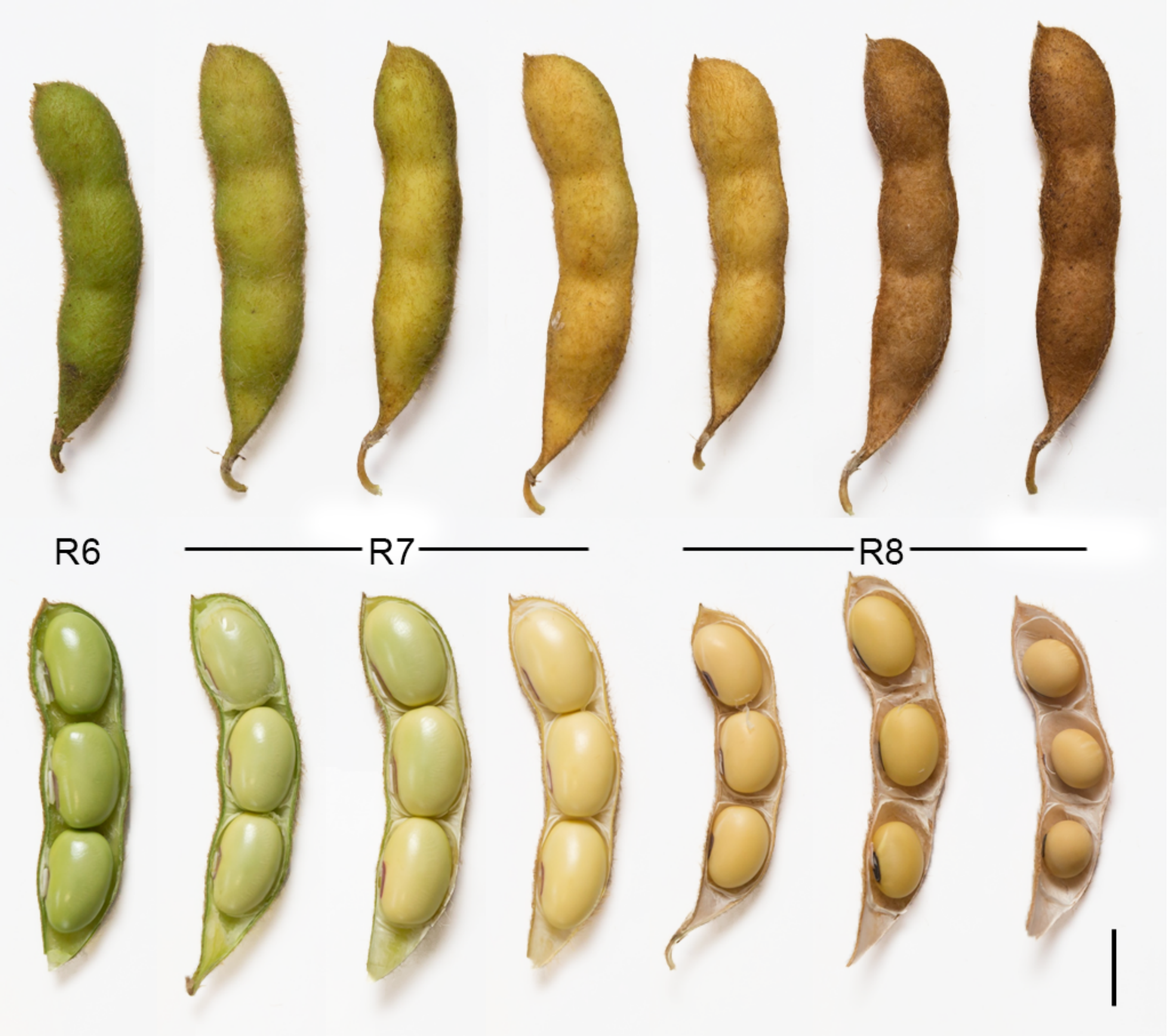

Figure S4. Pod and seed characteristics at the reproductive stages R6, R7 and R8, cultivar MG/BR 46. The black bar represents 1cm.

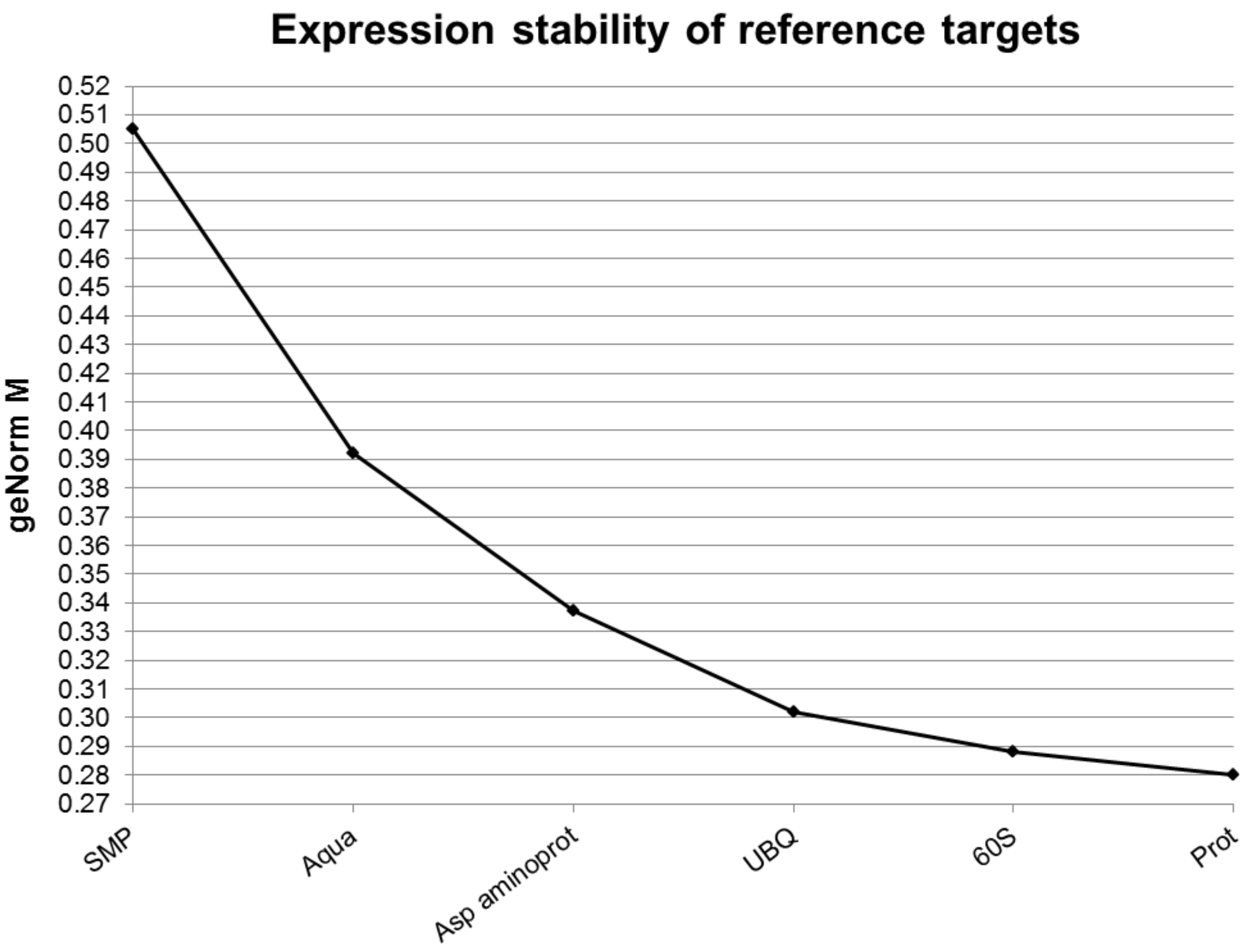

Figure S5. Expression stability of 6 reference genes for soybean seed research based on microarray expression data analyzed by geNorm.
